# From Cellulose, Shrimp and Crab Shells to Energy Storage EDLC Cells: The Study of Structural and Electrochemical Properties of Proton Conducting Chitosan-Based Biopolymer Blend Electrolytes

**DOI:** 10.3390/polym12071526

**Published:** 2020-07-09

**Authors:** Shujahadeen B. Aziz, Muhamad. H. Hamsan, Muaffaq M. Nofal, Saro San, Rebar T. Abdulwahid, Salah Raza Saeed, Mohamad A. Brza, Mohd F. Z. Kadir, Sewara J. Mohammed, Shakhawan Al-Zangana

**Affiliations:** 1 Hameed Majid Advanced Polymeric Materials Research Lab., Department of Physics, College of Science, University of Sulaimani, Qlyasan Street, Sulaimani 46001, Kurdistan Regional Government, Iraq; rebar.abdulwahid@univsul.edu.iq (R.T.A.); mohamad.brza@gmail.com (M.A.B.); 2 Department of Civil Engineering, College of Engineering, Komar University of Science and Technology, Sulaimani 46001, Kurdistan Regional Government, Iraq; 3 Institute for Advanced Studies, University of Malaya, Kuala Lumpur 50603, Malaysia; hafizhamsan93@gmail.com; 4 Department of Mathematics and General Sciences, Prince Sultan University, P. O. Box 66833, Riyadh 11586, Saudi Arabia; muaffaqnofal@gmail.com; 5 Department of Physics and Astronomy, University of Missouri-Kansas City, MO 64110, USA; ssawcc@mail.umkc.edu; 6 Department of Physics, College of Education, University of Sulaimani, Old Campus, Sulaimani 46001, Kurdistan Regional Government, Iraq; 7 Charmo Research Center, Charmo University, Peshawa Street, Chamchamal, Sulaimani 46001, Kurdistan Regional Government, Iraq; salah.saeed@charmouniversity.org; 8 Manufacturing and Materials Engineering Department, Faculty of Engineering, International Islamic University of Malaysia, Kuala Lumpur 50603, Gombak, Malaysia; 9 Centre for Foundation Studies in Science, University of Malaya, Kuala Lumpur 50603, Malaysia; mfzkadir@um.edu.my; 10 Department of Chemistry, College of Science, University of Sulaimani, Qlyasan Street, Sulaimani 46001, Kurdistan Regional Government, Iraq; sewara.mohammed@univsul.edu.iq; 11 Department of Physics, College of Education, University of Garmyan, Kalar 46021, Kurdistan Regional Government, Iraq; shakhawan.al-zangana@garmian.edu.krd

**Keywords:** polymer blend, XRD and FTIR, impedance study, TNM and LSV, CV and EDLC

## Abstract

In this study, solid polymer blend electrolytes (SPBEs) based on chitosan (CS) and methylcellulose (MC) incorporated with different concentrations of ammonium fluoride (NH_4_F) salt were synthesized using a solution cast technique. Both Fourier transformation infrared spectroscopy (FTIR) and X-ray diffraction (XRD) results confirmed a strong interaction and dispersion of the amorphous region within the CS:MC system in the presence of NH_4_F. To gain better insights into the electrical properties of the samples, the results of electrochemical impedance spectroscopy (EIS) were analyzed by electrical equivalent circuit (EEC) modeling. The highest conductivity of 2.96 × 10^−3^ S cm^−1^ was recorded for the sample incorporated with 40 wt.% of NH_4_F. Through transference number measurement (TNM) analysis, the fraction of ions was specified. The electrochemical stability of the electrolyte sample was found to be up to 2.3 V via the linear sweep voltammetry (LSV) study. The value of specific capacitance was determined to be around 58.3 F/g. The stability test showed that the electrical double layer capacitor (EDLC) system can be recharged and discharged for up to 100 cycles with an average specific capacitance of 64.1 F/g. The synthesized EDLC cell was found to exhibit high efficiency (90%). In the 1st cycle, the values of internal resistance, energy density and power density of the EDLC cell were determined to be 65 Ω, 9.3 Wh/kg and 1282 W/kg, respectively.

## 1. Introduction

Energy storage devices, such as lithium batteries, supercapacitors and fuel cells using liquid electrolytes, have attracted significant attention in recent years, owing to their ionic nature. However, there are several issues that still need to be solved, such as the release of harmful gases, the lack of safety and corrosive action [1]. It is difficult to use a harmless liquid in energy storage devices without any drawbacks. Designing a desirable device with a proper size and shape that fits liquid electrolytes is challenging [2]. This encourages scientists and researchers to work on the development of a safe and efficient solid polymer electrolyte (SPE). SPEs can provide satisfactory thermal stability, low weight, high flexibility, cost effectiveness and easy handling [3]. On the other hand, the harmful effects of plastic wastes on the environment are recognized to cause global warming and water pollution. Therefore, there is a special interest in the development of biodegradable and biocompatible natural polymers as SPEs [4]. This is due to their abundance, biocompatibility, biodegradability and cost effectiveness [5]. These favorable properties have made scientific circles extensively utilize natural polymers in polymer electrolyte-based devices [6]. There are many natural polymers, including starch, CS, carboxymethyl cellulose (CMC), MC and rubber that can be used in the synthesis of SPEs [5,6]. In this study, CS and MC were used as natural polymers. CS is often extracted from crustaceans (crabs, lobsters, crayfish, shrimp, krill and barnacles) and has a chemical structure of β-(1→4)2-amino-2-deoxy-D-glucose-(D-glucosamine) [4], while MC is obtained from mixing alkali-based cellulose with methyl chloride. The chemical structure of MC comprises a 1,4 glycosidic bond [7]. The reduced ion mobility in SPE matrices has led numerous research groups to develop different approaches that have improved the ambient conductivity. Two common approaches which are widely addressed are the blending of two polymers and using a variety of salts [8,9,10,11,12,13]. Polymer blending is regarded as a promising technique to upgrade the properties of individual polymer constituents. Many new and enhanced characteristics can be achieved through the polymer blending technique, such as relatively high ionic conductivity, flexibility, transference number and thermal stability [14]. Recently, polymer electrolytes assembled from biopolymer attracted the attention of many research groups due to their availability for a wide range of applications in electrochemical devices [2,3,4,8,10,11,12]. Both CS and MC are known to contain functional groups with lone pair electrons that assist ion transport within their matrixes. This is due to the fact that the lone pair electrons within their structure can serve as complexation sites for the ions. 

The preparation of proton (H^+^)-conducting SPEs is usually involves mixing strong inorganic acids or ammonium salts. For instance, sulfuric acid (H_2_SO_4_) and phosphoric acid (H_3_PO_4_) are the two commonly used inorganic acids. However, the main drawback of these inorganic acids is their chemical degradation when mixed with SPEs, which leads to incompatibility with practical applications [15]. Therefore, the ammonium salts are usually utilized to obtain a proton-conducting SPE with a relatively high ionic conductivity and thermal stability. The continuous interactions of the charge carriers with the available functional group then generates motion of the polymer chain segments and thus makes the polymer more conductive [16]. Radha et al. have documented a conductivity value of 6.9 × 10^−6^ S/cm for the polyvinyl alcohol (PVA)–ammonium fluoride (NH_4_F) system [17]. Additionally, the enhancement in the dielectric behavior of PVA with the presence of NH_4_F was also addressed. Another research study has also recently reported a relatively high conductivity of 6.40 × 10^−7^ S/cm for the MC–NH_4_F system at room temperature [5]. Doping NH_4_F into the CS–dextran system also resulted in a high value of conductivity, up to 10^−3^ S/cm [18].

Electrical double layer capacitors (EDLCs) are one of the promising electrochemical energy storage devices that fulfill the requirements of high power application with fast charge–discharge cycles. The energy storage mechanism in these devices involves charge accumulation on the carbon electrode surface at the interfacial region in the form of potential energy [19]. EDLCs feature a fantastic high power density, long cycle life, fast charge–discharge rate and simplistic fabrication procedure [20]. In EDLC devices, various types of carbon have been used as electrode materials, for instance, graphite [21], aerogel [22], carbon nanofibers [23] and activated carbon [24]. The most used one is activated carbon, which is almost an ideal active material that is defined by a high surface area, satisfactory electronic conductivity and cost effectiveness [25]. Based on an earlier study, ammonium salts have been shown to exhibit reasonable proton donor behavior if incorporated into polymer matrices [26]. In order to enhance the performance of biodegradable-based EDLC devices to meet the industrial level, various polymer blended electrolytes with different dopant salts were studied. An extensive literature survey revealed that the effect of NH_4_F salt concentration on the conductivity of a CS:MC blended system and its use in EDLC devices had not already been investigated. Thus, for this work, firstly, systems of CS:MC incorporated with various concentrations of NH_4_F were examined through electrical and structural analyses. Then, the relatively highest conducting SPBE film was utilized as the electrode separator in an EDLC device application.

## 2. Experimental Part

### 2.1. Materials and Sample Preparation

In this work, CS with a relatively high molecular mass of around 310,000 to 375,000 g/mol was used, along with MC and NH_4_F in the fabricating of SPBE systems. All the materials were supplied by Sigma-Aldrich Corporation (Missouri, MO, USA). Firstly, for the preparation of CS:MC polymer blend electrolytes, two separated solutions of CS and MC with percentages of 70 wt.% and 30 wt.%, respectively, were dissolved in 40 mL of 1% acetic acid. They were stirred for 3 hrs at room temperature. Based on previous work [27], this ratio of CS and MC was shown to be optimal in the preparation of CS:MC polymer blend electrolytes. Then, both solutions of CS and MC were mixed with continuous stirring for 2 hrs in order to obtain a final homogeneous blended solution. Subsequently, with continuous stirring, different portions of NH_4_F, ranging from 10 to 40 wt.% in steps of 10 wt.%, were added separately to a series of blended solutions to obtain CS:MC:NH_4_F electrolytes. The final solutions were then poured into Petri dishes to cast films at ambient temperature. For further drying, the formed films were then transferred into a desiccator to achieve solvent-free films. The obtained polymer blend electrolyte samples were coded as CMCF0, CMCF1, CMCF2, CMCF3 and CMCF4 for CS:MC doped with 0, 10, 20, 30 and 40 wt.% of NH_4_F, respectively.

### 2.2. Structural and Impedance Analyses

XRD measurements were performed to study the structural properties of the samples, using a Siemens D5000 X-ray diffractometer (1.5406 Å) (Bruker AXS GmbH, Berlin, Germany). The acquisition process comprised scanning the 2θ angle continuously from 5° to 80° (resolution = 0.1°). FTIR (FT-IR, Spotlight 400 Perkin-Elmer spectrometer, Waltham, MA, USA) was conducted in the range 450 to 4000 cm^−1^ with a 1 cm^−1^ resolution. Electrical properties of the samples were studied by means of EIS (HIOKI 3532–50 LCR Hi-TESTER) (Hioki, Nagano, Japan) in the frequency range of 50 Hz to 5 MHz. For the purpose of electrical characterizations, the samples were sandwiched between two stainless steel electrodes. Based on the results obtained in EIS measurements and the sample dimensions, the conductivities of the samples were determined, using the following equation:

(1) $$\sigma_{dc}=\left(\frac{1}{R_{b}}\right)\times \left(\frac{t}{A}\right)$$

where *R_b_* is the bulk resistance of the sample, *t* is the film thickness and *A* is the surface area of the film. 

### 2.3. Transference Number Measurement (TNM) and Linear Sweep Voltammetry (LSV) Studies 

**Figure 1:**
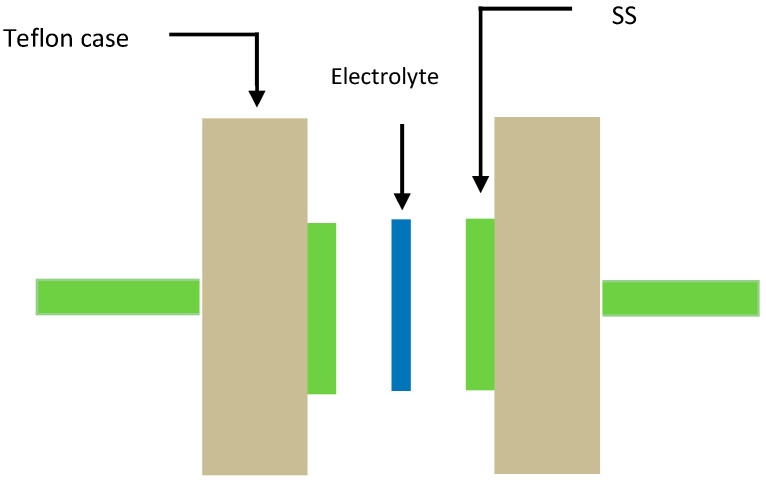
Schematic appearance of the cell used for the LSV and TNM measurements.

To use polymer blend electrolytes in applications, it is important to study their TNM and LSV measurements. Through TNM measurements, one can verify the ion dominancy in the conduction process. LSV measurements were used to investigate the sample electrolytes’ potential stability. The TNM measurements were performed by using a V & A Instrument DP3003 digital DC power supply with 0.20 V (V & A Instrument, Shanghai, China). LSV measurements were also carried out by using a Digi-IVY DY2300 Potentiostat at a sweep rate of 50 mV s^−1^ (Neware, Shenzhen, China). The cell used in both TNM and LSV measurements was composed of two stainless steel (SS) disks and the highest conducting SPBE film. The cell was then mounted in a Teflon holder, as shown in Figure 1.

### 2.4. EDLC Preparation 

For the fabrication of an EDLC device, electrodes composed of polyvinylidene fluoride (PVdF), activated carbon and carbon black materials, were used. Under medium stirring, 0.5 g of PVdF was dissolved in 15 mL of N-methyl pyrrolidone (NMP) to obtain an NMP–PVdF solution. The activated carbon and carbon black materials were dry mixed for 15 min by using a planetary ball miller (XQM-0.4) with a rotational speed of 500 r/min. The obtained powders were composed of 3.25 g and 0.25 g of activated carbon and carbon black materials, respectively. The powder was then poured into the NMP–PVdF solution and stirred to dissolve completely. Then, the mixture was cast on an aluminum foil with a doctor blade technique to obtain a thick black solution. Subsequently, the folded solution was heated at 60 °C in an oven for a certain time to obtain a dried state. The obtained dried bulk electrode was then cut into a circle with an area of 2.01 cm^2^. In the final step of the EDLC cell preparation, the relatively high conducting electrode film was sandwiched between two carbon electrodes and packed in CR2032 coin cells. The fabricated EDLC cell was then fixed over the Teflon holder for further testing. Cyclic voltammetry of the EDLC was performed at 10 mV s^−1^ and charged up to 0.90 V, by using a Digi-IVY DY2300 Potentiostat (Neware, Shenzhen, China). Furthermore, the charge–discharge profiles of the EDLC were also examined using a Neware battery cycler (Neware, Shenzhen, China) with a current density of 0.2 mA cm^−2^.

## 3. Result and Discussion

### 3.1. XRD and FTIR Study

**Figure 2:**
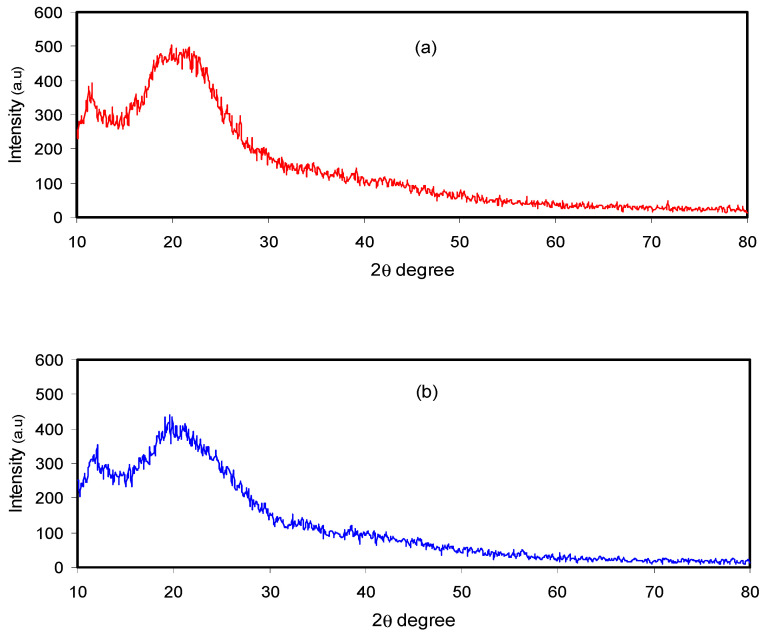

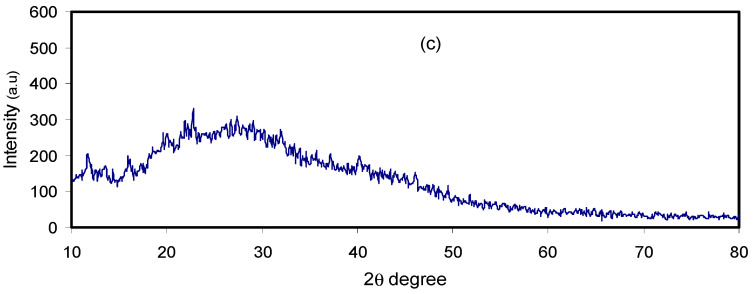
XRD pattern for (**a**) pure CS:MC, (**b**) CMCF2 and (**c**) CMCF4 blend electrolytes.

**Figure 3:**
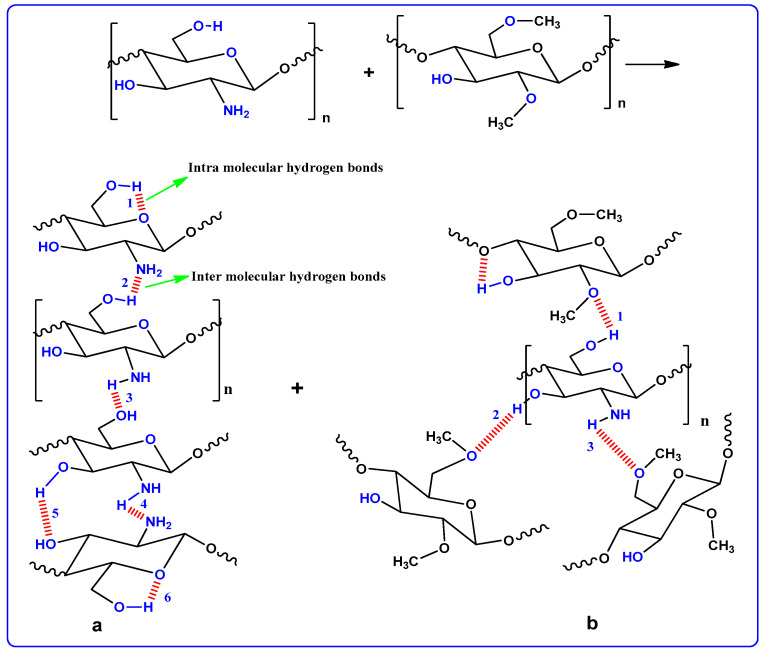
CS and MC polymer hydrogen bonding presentation; (**a**) hydrogen bonding through CS polymer and (**b**) hydrogen bonding CS:MC polymer blends.

Figure 2 shows the XRD pattern of the pure and doped CM:MC samples. Previous studies have illustrated that pure MC displays broad humps and several weak peaks at 2θ = 8° and 21° [28]. It has also been revealed that CS in its pure state, which has a predominant crystalline phase, is characterized by two obvious peaks at 14.50° and 20.90° as a result of inter- and intra-hydrogen bonding [29]. It is obvious in Figure 2a that the XRD peaks due to pure CS almost disappeared and only a hump can be seen. This indicates that the technique of polymer blending is a novel approach to overcome the crystalline phases. This is due to the formation of hydrogen bonding between MC and CS matrices. It is well defined that hydrogen bonding is based on the interaction between electron-deficient hydrogen and a high electron density region. In fact, hydrogen bonding (H-bonding), as an intermolecular interaction, can be expressed as X–H…Y, where X and Y are electronegative elements and Y possesses one or more lone electron pairs; in other words, X and Y are F, O and N atoms, respectively [30]. It is evident from the molecular structure of MC (see Figure 3) that the monomer of MC contains O atoms, which enables it to build H-bonding. Clearly, hydrogen bonding as a secondary force is much weaker than the primary bond within the molecules, such as covalent bonds and other polar bonds, but far stronger than the van der Waals interaction [30]. It is notable from Figure 3a that about six hydrogen bonds can be formed between CS monomers; meanwhile, only three intermolecular hydrogen bonds can be developed between the CH and MC monomer, as depicted in Figure 3b. Therefore, the blended samples showed a smaller number of hydrogen bonding sites, which resulted in the reduction of the degree of crystallinity. The XRD peak broadness of the CS:MC blend sample pinpoints that the inter-chain spacing in the blended sample became larger than that of the individual polymers. The expanded inter-chain spacing in the blend simplifies the dipole reorientation to the applied field due to high amorphous content. The addition of NH_4_F to the blend electrolytes (BEs) caused a clear reduction in the intensity of XRD peaks. Interestingly, upon the addition of 40 wt.% of NH_4_F, the lowest peak intensity was recorded. It is evidenced that the broad peak at 20.25° (see Figure 2c) emphasizes the amorphous nature of the system. The characteristic feature of the amorphous nature of a polymer body is a broad peak in the form of a hump. Increasing salt in such a polymer system caused a relative reduction in the intensity of the broad peak between 11° and 27.21°, thus increasing the amorphous structure of the BE system [26]. Therefore, the XRD pattern can certainly be used to show the amorphous characteristic of the samples, which was reflected in the gradual decrease in intensity with peak broadening due to the addition of NH_4_F salt. It is also distinct that the dissimilarity in the intensity and sharpness of the XRD peaks of the polymer electrolytes after salt addition can be a good confirmation of strong interactions between the polymer and the inorganic salt [29].

**Figure 4:**
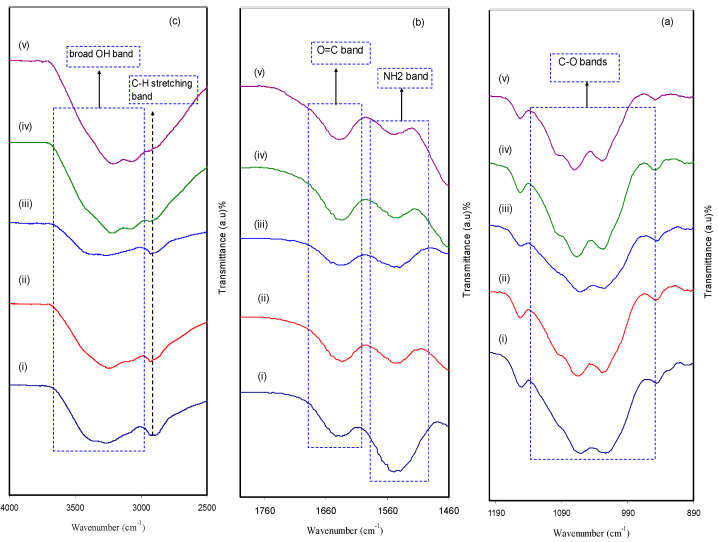
FTIR spectrum of (**i**) CMCF0, (**ii**) CMCF1, (**iii**) CMCF2, (**iv**) CMCF3 and (**v**) CMCF4 in the range (**a**) 890 cm^−1^ to 1190 cm^−1^, (**b**) 1460 cm^−1^ to 1760 cm^−1^ and (**c**) 2500 cm^−1^ to 4000 cm^−1^.

FTIR is considered as an effective technique to deal with a new compound, in terms of both structure and composition, that forms during a chemical reaction. The extent of interaction between CS and MC in the BPE system was confirmed via the FTIR technique. Figure 4a–c shows the FTIR spectra of CS:MC biopolymer electrolytes in the wave number range of 4000−890 cm^−1^. It is motivating to observe both position shifting and intensity variation of the bands, which are considered as evidence of the existence of particular functional groups in pure CS:MC and CS:MC:NH_4_F electrolyte systems. A more important observation is the confirmation of the presence of heteroatoms (e.g., O and N) with lone pair electrons in a desired fabricated polymer host as electrolytes [31]. The appearance of a strong peak at around 2900 cm^−1^ is ascribed to the C-H stretching modes, as shown in Figure 4c [32,33], and its intensity decreased with rising salt concentration. It is also seen from the same figure that the CS polymer is characterized by a single -NH_2_ group and a couple of -OH groups in the repeating unit [34]. The doping process substantially affected the -OH stretching broad peak at 3359 cm^−1^ in Figure 4c [32]. The strong interaction between dopant salt and the CS:MC host blended polymer can be identified from both peak shifting and intensity changes. Based on earlier work [34], the presence of vibrational frequency peaks of -NH_2_, O=C-NHR and –OH are considered as the characteristic FTIR spectra of MC and CS polymers. It is clear from Figure 4b that the shifting occurred towards lower wave numbers in the bands of amino NH_2_, O=C-NHR and OH groups, confirming a strong interaction between the NH_4_F dopant salt and the CS:MC host blended polymer.

In Figure 4a, a peak at 1055 cm^−1^ appeared as a result of the antisymmetric stretching of an asymmetric oxygen bridge in the cyclohexane ring and a range from 1150 to 1000 cm^−1^ is attributed to the C-O-C bond [28]. It is observable that the intensity of the peaks decreased and to some extent shifting occurred as a consequence of the increment of NH_4_F concentration. These certify that there is a strong interaction between the polymer body and amine salt via coordination bonds and thus confirms the complexation [35]. The addition of NH_4_F salt provides cations that attract oxygen atoms at the C-O-C ether group in the identical polymer to produce polymer salt complexes [28]. In the current polymer salt system, the NH^+^_4_ ion from NH_4_F coordinates to both the O atom of the ether group and the hydroxyl group in the CS and MC host polymer blend. These interactions prove the occurrence of protonation in the present electrolytes. It is clearly verified in the shifting of hydroxyl, ether, C=O and -NH_2_ [5].

### 3.2. Impedance Study

**Figure 5:**
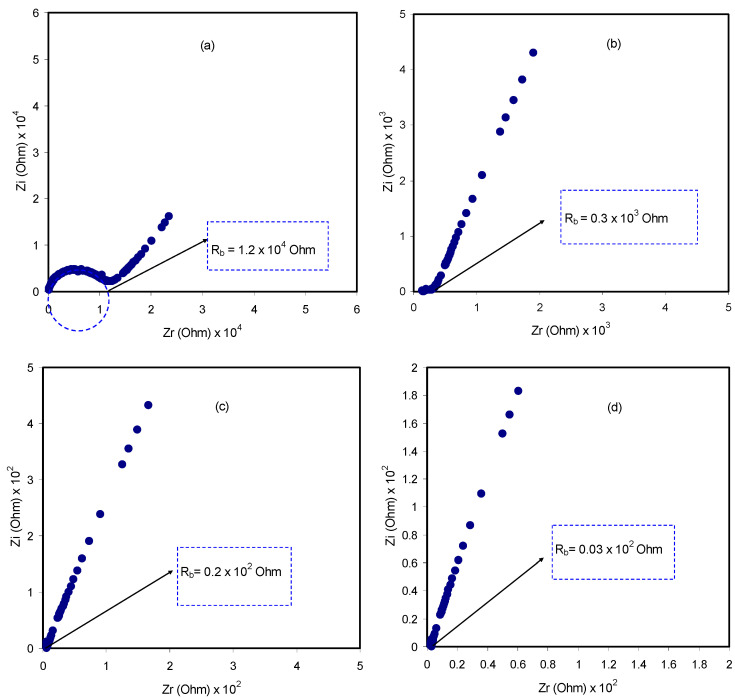
Impedance plots of (**a**) CMCF1, (**b**) CMCF2, (**c**) CMCF3 and (**d**) CMCF4 blended films at ambient temperature.

**Figure 6:**
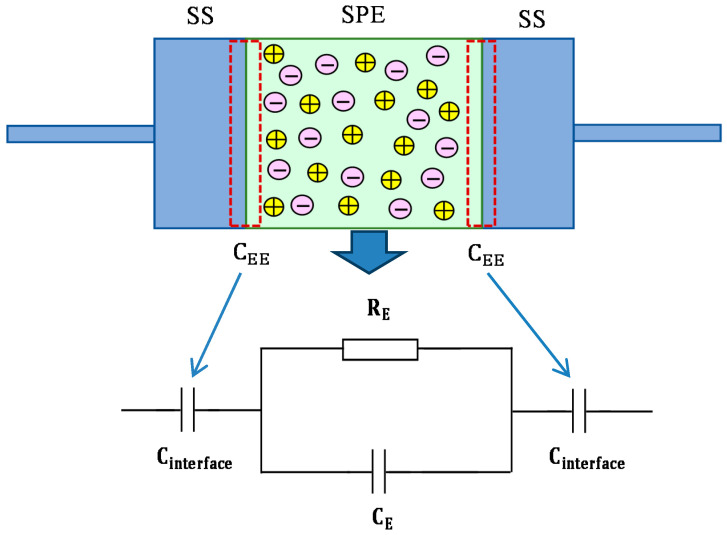
Schematic illustration of electrical equivalent circuits (EECs) for impedance plots consisting of a high-frequency semicircle and low-frequency tails.

**Figure 7:**
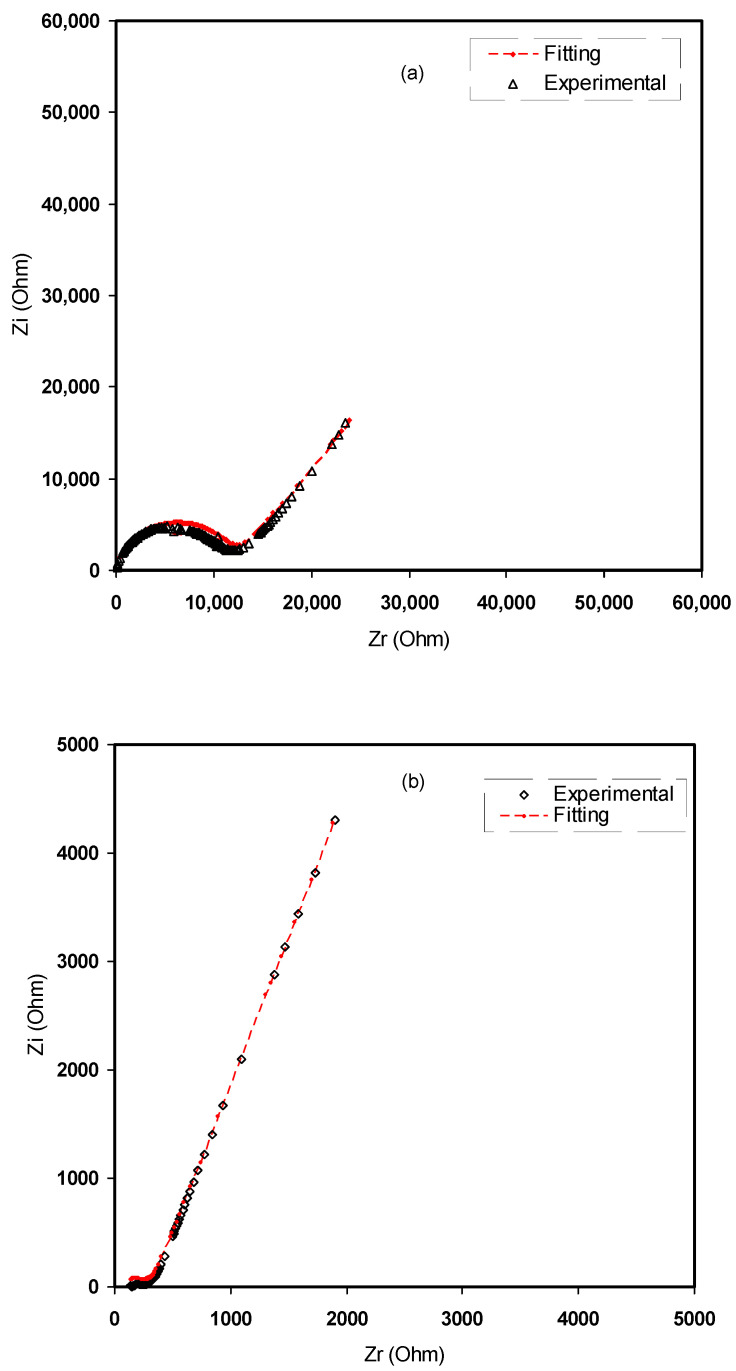

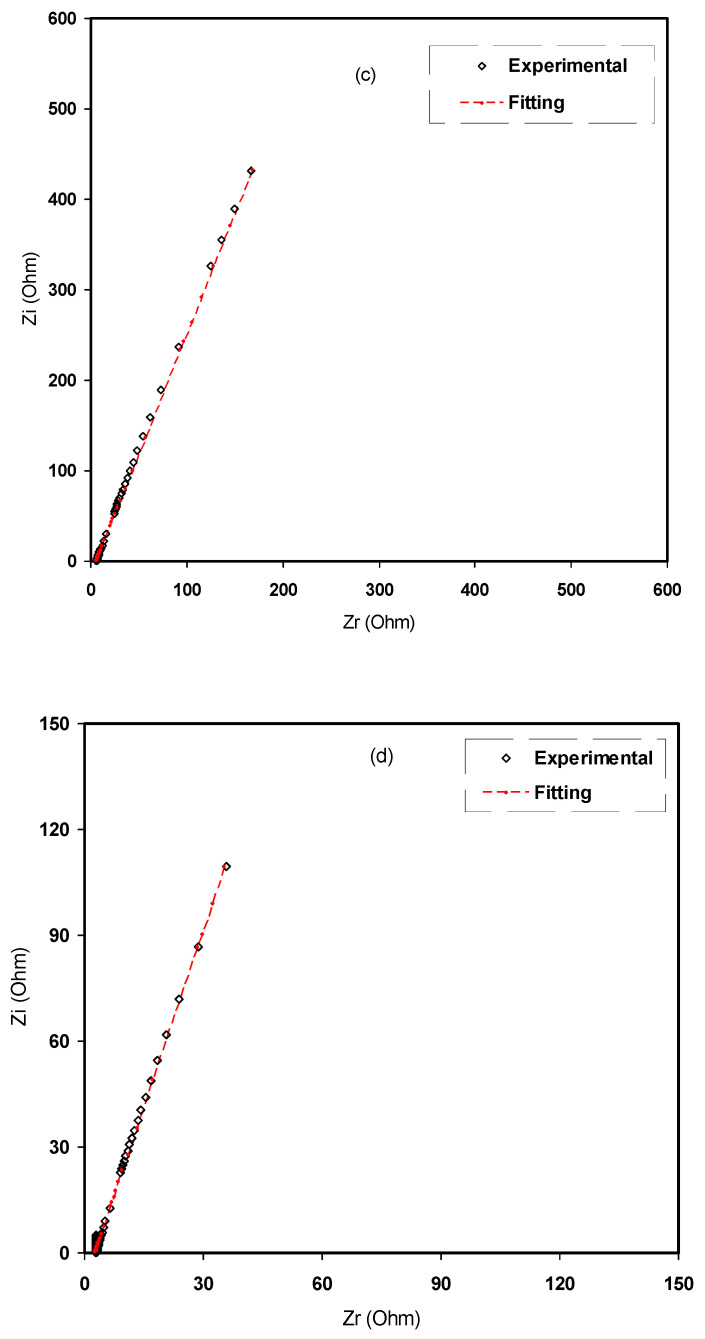
Experimental impedance and EEC fitting plots for (**a**) CMCF1, (**b**) CMCF2, (**c**) CMCF3 and (**d**) CMCF4 blended films at ambient temperature.

The impedance spectra of the blend electrolyte films at ambient temperature are shown in Figure 5a–d. The semicircle at the high frequency region can be related to the parallel combination of the bulk electrolyte resistance (R_E_) and the bulk electrolyte capacitance (C_E_), owing to the migration process of proton ions and the immobilized state of polymer chains, respectively. Interestingly, the semicircle diameter was lessened with increasing salt concentration. This implies that the relaxation of ions occurred at different times [36]. The electrode/electrolyte capacitance (C_EE_) produced by the accumulated double-layer ions at the electrode/electrolyte interface (i.e., the low frequency spike region) is represented by another capacitor in series with the parallel combination of a resistor and capacitor corresponding to the high frequency semicircle. The schematic illustration of EEC for Figure 5a,b is shown in Figure 6. To confirm our interpretation, the experimental impedance data were simulated with EECs, as can be seen in Figure 7. It is worth noting that at a high frequency region, the semicircle completely disappeared for 30 and 40 wt.% of NH_4_F salt concentrations, as clearly shown in Figure 5c,d. This suggested that only the resistive component of the obtained impedance spectra is responded to by the polymer host body [27]. In this case, the resistor of the blend electrolyte film in series with the capacitor of the double-layer capacitances represents the electrical behavior of the system. The conductivity improvement upon the addition of 40 wt.% of NH_4_F can be attributed to the increase in the number of mobile charge carriers. Moreover, the amorphous nature of the polymer electrolyte could have a vital role. Consequently, it results in an inferior energy barrier and facilitates the ion transport [35]. It is self-evident that the ionic conductivity of an electrolyte depends on both the number and mobility of ions, as can be seen from the following equation [37]:

(2) σ=∑ηqμ

where the carrier density is denoted as *η*, elementary charge is symbolized by *q* and *μ* is the mobility. The literature confirmed that in a polymer–ammonium salt system, the charge-carrying species is an H^+^ ion that is offered by an ammonium ion [27]. The most general theory of proton conduction is structure diffusion, which is known as the Grotthuss mechanism, where ion exchanging occurs between the complexed sites [38]. Proton conduction by the Grotthus mechanism states that protons jump over the complexing sites, leading to the creation of a vacant site followed by reorientation to occupy the vacant site [27]. Equation (1) was used to calculate the DC conductivity of the pure CS:MC and CS:MC:NH_4_F electrolyte samples at room temperature. Table 1 lists the DC conductivities of the samples. It is noteworthy that the DC conductivity increased from 7.16 × 10^−10^ S cm^−1^ for pure CS:MC to 7.34 × 10^−4^ S cm^−1^ for CS:MC incorporated with 40 wt.% of NH_4_F. Previous studies have confirmed that polymer electrolytes with high DC conductivity, ranging from 10^−5^ to 10^−3^ S cm^−1^, can be crucial for electrochemical device applications, including batteries and EDLCs.

**Figure 8:**
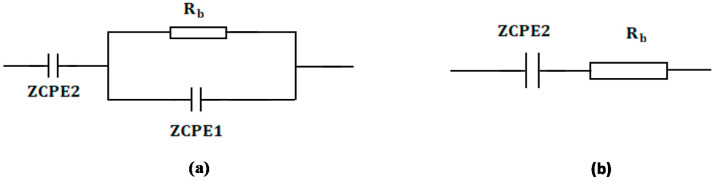
Schematic illustration of the EECs for (**a**) parallel combination of a resistor and capacitor in series with another capacitor and (**b**) series combination of a resistor and capacitor. The resistor is represented by the symbol 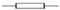 and the capacitor is represented by 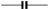.

From the fitting and analysis of experimental spectra using the EEC technique, one can easily deal with the mechanism of the system under study [39]. The experimental impedance plots with EEC modeling for all the electrolyte samples are shown in Figure 7a–d. This model guides us to calculate and understand the electrical properties of solid-based electrolyte polymers. A three-component equivalent circuit reflects the experimental impedance plots. To be precise, the three principal elements are ZCPE1 (constant phase, electrode/electrolyte capacitance, C_interface_), ZCPE2 (another constant phase, electrolyte capacitance, C_E_) and a bulk resistance R_b_ for the SPE (bulk electrolyte resistance, R_E_) (see Figure 8a). Two elements, *R_b_* and ZCPE1, are obtained in the high frequency region; in other words, both respond in the high frequencies, whereas the low frequency spike region is linked to ZCPE2. The EECs corresponding to Figure 7a,b are shown in Figure 8a. The ZCPE1 is the response of the double-layer capacitance formed at the interface region between the electrodes and the SPE [40]. It is possible to derive ZCPE’s impedance as [41]:

(3) $$Z_{CPE}=\frac{1}{C\omega^{p}}\left[\cos\left(\frac{\pi p}{2}\right)-i\sin\left(\frac{\pi p}{2}\right)\right]$$

where CPE is constant phase element, capacitance is represented by C, the angular frequency is denoted by *ω* and *p* is related to the departure of the plot from the vertical axis in complex impedance plots. It is worth mentioning that CPE is the acronym most commonly used instead of capacitor in the context of an EEC model. It is appealing to see that in such a system, the capacitor element in the circuit is replaced by CPE, where it is a capacitor system in an ideal or pure capacitor. Stated differently, this implies that the system is a semi-capacitor [40,42], which is the nature of a capacitor in the electrolyte/electrode system. This is not the case to recognize an ideal capacitor system in the existing experimental impedance spectra. For the case of expressing the real (Zr), imaginary (Zi) and complex impedance (Z*) values in the equivalent circuit, it can be formulated in a mathematical expression in the following way [41]:

(4) $$Z_{r}=\frac{R_{b}C_{1}\omega^{p1}\cos\left(\frac{\pi p_{1}}{2}\right)+R_{b}}{2R_{b}C_{1}\omega^{p}\cos\left(\frac{\pi p}{2}\right)+R_{b}{}^{2}C_{}{}^{2}\omega^{2p}+1}+\frac{\cos\left(\frac{\pi p_{2}}{2}\right)}{C_{2}\omega^{p2}}$$

(5) $$Z_{i}=\frac{R_{b}C_{1}\omega^{p1}\sin\left(\frac{\pi p_{1}}{2}\right)}{2R_{b}C_{1}\omega^{p}\cos\left(\frac{\pi p}{2}\right)+R_{b}{}^{2}C_{}{}^{2}\omega^{2p}+1}+\frac{\sin\left(\frac{\pi p_{2}}{2}\right)}{C_{2}\omega^{p2}}$$

where *R_b_* is the bulk resistance. Based on Equations (4) and (5), the experimental impedance plots are well simulated, as shown in Figure 7, and the EECs are presented in Figure 8. From the impedance spectra, it is obviously seen that the semicircle size dropped at the high frequency region as the concentration of salt increased (20 wt.% of NH_4_F). Figure 7a,b exhibits a model comprising the incomplete semicircle, from which one can extract the value of *R_b_* that is parallel with the CPE element and series with another CPE relating to a low frequency tail, as shown schematically in Figure 8a. Predictably, the incomplete semicircle at 30 wt.% and 40 wt.% of NH_4_F totally disappeared, as shown in Figure 7c,d. This indicates the possibility of the resistive behavior of SPEs and the CPE component in series, as shown schematically in Figure 8b. The equivalent circuit element parameters of the blend electrolytes are shown in Table 2. The logical explanation for this result, where the semicircle disappeared at the high frequency region in the spectra, is explained by the entire conductivity attributed to a huge ion migration [43]. In this case, the values of *Z_r_* and *Z_i_* are correlated to the EEC and can be expressed mathematically as follows:

(6) $$Z_{r}=\frac{\cos\left(\frac{\pi p}{2}\right)}{C\omega^{p}}+R_{b}$$

(7) $$Z_{i}=\frac{\sin\left(\frac{\pi p}{2}\right)}{C\omega^{p}}$$

### 3.3. EDLC Characteristics

TNM and LSV Study

**Figure 9:**
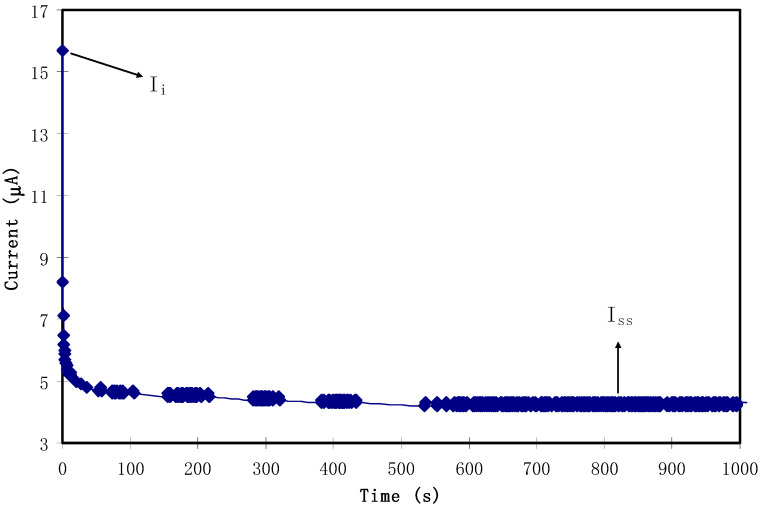
TNM plot for the highest conducting solid polymer electrolyte (SPE) with a polarization voltage of 0.2 V.

To study total conductivity in SPBEs, TNM analysis was determined and the dominancy of ions to the total conductivity was verified. It was also proven that electrons partly contributed to the overall conductivity. Therefore, both electronic (*t_e_*) and ionic (*t_i_*) transference numbers can be obtained via:

(8) $$t_{i}=\frac{I_{i}-I_{s}}{I_{i}}$$

(9) $$t_{i}=1-t_{e}$$

where *I_i_* is the current at the initial stage and *I_s_* is the current at the constant stage. Figure 9 displays the TNM plot for the highest conducting SPE. From the procedure, as the cell was disturbed by the working voltage of 0.2 V, the value of *I_i_* was obtained at 15.7 A. The high value of the current at the initial stage was assigned to both ions and electrons as charge-carrying species. As the procedure continued, a dramatic drop in the current was seen before reaching a constant value of 4.2 A. Obviously, this decrease in the initial total current is ascribed to the depletion of the ionic species in the bulk electrolyte and became constant in the completely depleted state [43]. This phenomenon is explained on the basis of the ion-blocking effect at the stainless-steel electrodes. Kufian et al. [44] clarified this behavior and stated that, as polarization occurs in the cell, a constant current stage reaches its value and the cause of the remaining current flow is related to electrons alone. The values of *t_e_* and *t_i_* were determined to be 0.27 and 0.73, respectively. This finding is of major importance, since it clarifies the main contribution of ions compared to electrons in the total conductivity. These data results are in good harmony with those reported for the carboxylmethylcellulose–NH_4_F system by Ramlli and Isa [45]. Kyle et al. [46] recommended that the high value of the ionic transference number verifies to a large extent that the SPE behaves as an ionic conductor.

**Figure 10:**
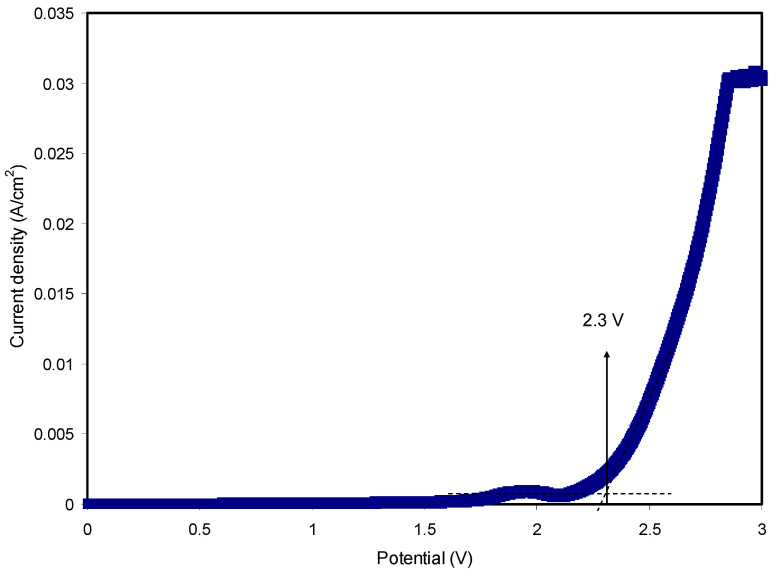
LSV plot for the highest conducting SPE with a scan rate of 50 mV/s.

Among a number of characteristics of SPEs, electrochemical stability is critical, and from this one can decide the viability of SPEs in electrochemical devices. In this work, the potential window extended to ~1 V [47]. Figure 10 exhibits the LSV for the relatively highly conducting SPE. Electrolytes used as electrode separators in EDLC devices will be subjected to a continuous process of rapid charge–discharge. During the charging process, a high voltage will be produced and the electrolyte film will breakdown. Thus, it is crucial to charge the EDLC to a potential value well below the breakdown voltage. Within the potential range from 0 to 1.7 V, there was no significant current change as the potential swept. Despite the current rising beyond 1.7 V, it was not considerable. As the potential exceeded 2.3 V, the current rose significantly, indicating electrolyte decomposition at the surface of the inert electrodes [48]. Remarkably, it is realized that the CS:MC:NH_4_F system is stable electrochemically within the specified potential range. Previously reported work [49] for an NH_4_F-based CS:dextran system found an electrochemical stability up to 1.7 V. Therefore, the CS:MC:NH_4_F system can be utilized in EDLC fabrication.

### 3.4. EDLC

**Figure 11:**
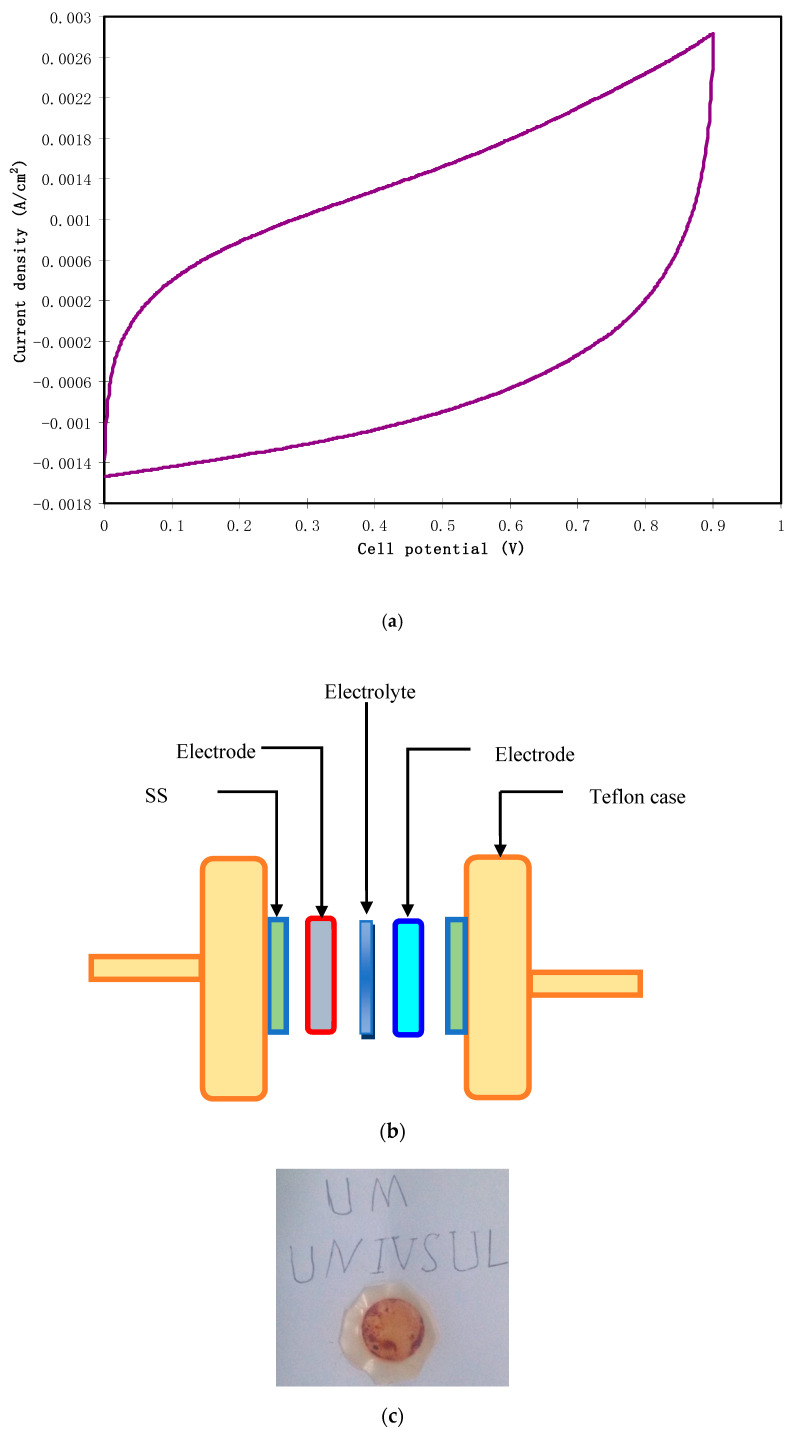
(**a**) CV measurement for the highest conducting SPE at 10 mV/s from 0 to 0.9 V, (**b**) schematic diagram of the CV measurement unit for the fabricated electric double-layer capacitor (EDLC) cell, and (**c**) realist image of the highest conducting SPE when reaching the breakdown voltage.

To investigate capacitive behavior of an EDLC, it is the preliminary test to record cyclic voltammetry (CV). Figure 11a highlights the CV of the assembled EDLC at a sweep rate of 10 mV/s. The schematic diagram for CV measurement is shown in Figure 11b and the realist image of the prepared electrolyte after the LSV test is presented in Figure 11c. It is noticed that the shape of the CV is almost rectangular with the absence of any redox peaks. This is a good sign of the pure EDLC (completely capacitor) and there was no signature for the existence of pseudocapacitors in the energy storage system [50]. Therefore, the non-Faradaic charge storage comprises ion adsorption and accumulation at the interfacial region rather than an intercalation/deintercalation process. More clearly, both electrons and ions accumulate at the interface of electrodes and electrolyte, respectively. The charge accumulation in this region makes a double-layer charge regime in the form of potential energy [51]. Typically, an ideal capacitor manifests in a perfect rectangular shape of CV. The non-ideal rectangular shape of our CV might be caused by both internal resistance and electrode porosity [52]. To analyze the obtained results, it is calculable to find the specific capacitance (*C_spe_*) of the assembled EDLC from the CV via the following equation:

(10) $$C_{spe}=\int_{V_{i}}^{V_{f}}\frac{I\left(V\right)dV}{2mv\left(V_{f}-V_{i}\right)}$$

where *I*(*V*)*dV* is the area of the CV which is determined using Origin 9.0 software through the integration function. The chosen *V_i_* and *V_f_* in the present work are 0 V and 0.9 V, respectively, and *m* and *v* are the mass of used active material and sweep rate, respectively. The value of *C_spe_* extracted from the CV was 58.3 F/g. This value is comparable to that obtained from the charge–discharge graph.

**Figure 12:**
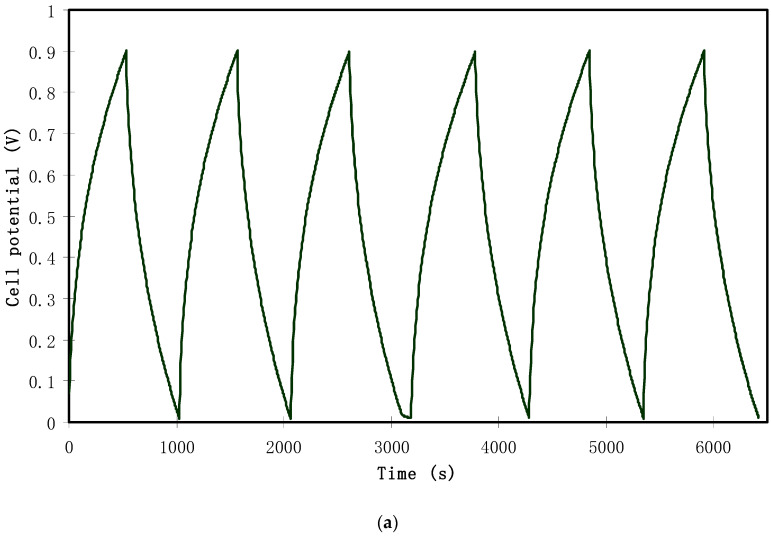

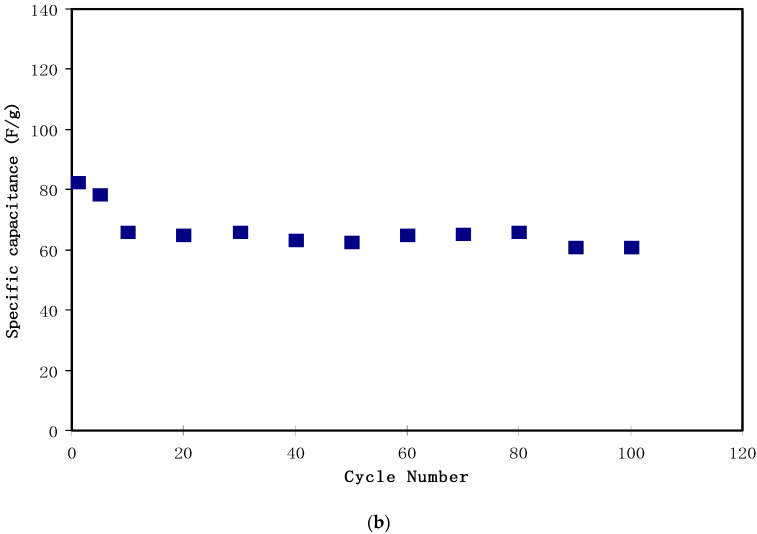
(**a**) Charge–discharge profile for the assembled EDLC at 0.2 mA/cm^2^, (**b**) specific capacitance of the assembled EDLC for 100 cycles.

At a current density of 0.2 mA/cm^2^, the rechargeability of the assembled EDLC is depicted in Figure 12a. From the figure, it can be seen that the plot is approximately linear in a triangular shape, which is a capacitive characteristic of the EDLC [53]. The results from the galvanostatic technique are in a high harmony with those from the capacitive CV analysis, confirming the capacitive behavior. It is straightforward to calculate specific capacitance (*C_s_*) from the slope (*s*) of the charging and discharging curve using the following relationship:

(11) $$C_{s}=\frac{i}{sm}$$

where *i* is the applied current, which was 0.4 mA in the present work.

Figure 12b presents *C_s_* for the complete 100 cycles. *C_s_* was 82.3 F/g for the 1st cycle. The relatively high value of *C_s_* in the initial few cycles was caused by the rapid development of the potential double layer due to electron and ion accumulation at the electrode and the electrolyte interfacial region. The value dropped to 78.4 F/g in the 5th cycle. This decline in specific capacitance value with increasing cycle number might be caused by ion association to form ion pairs or ion aggregation, which blocked the migration of free ions towards the carbon electrode [54]. Then, the value of *C_s_* stabilized from the 10th cycle to the 100th cycle with an average *C_s_* value of 64.1 F/g. This value was nearly the same as that obtained from the CV analysis. The study of the EDLC of various active materials and their corresponding specific capacitances are presented in Table 3.

**Figure 13:**
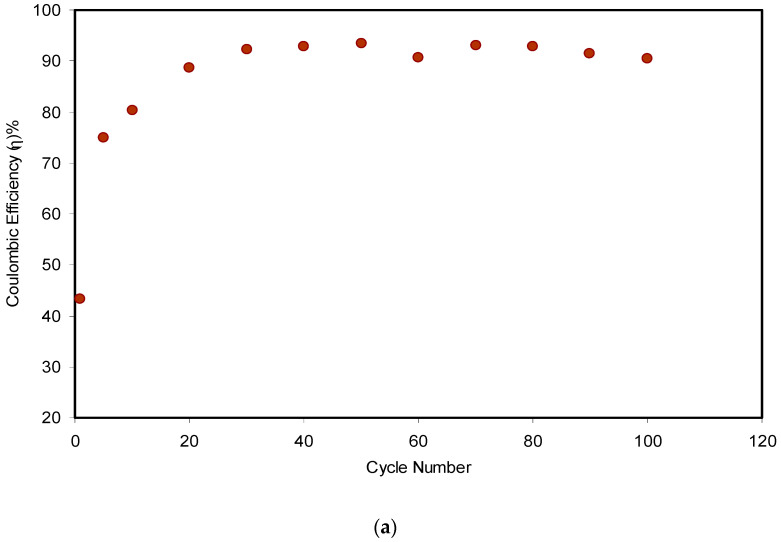

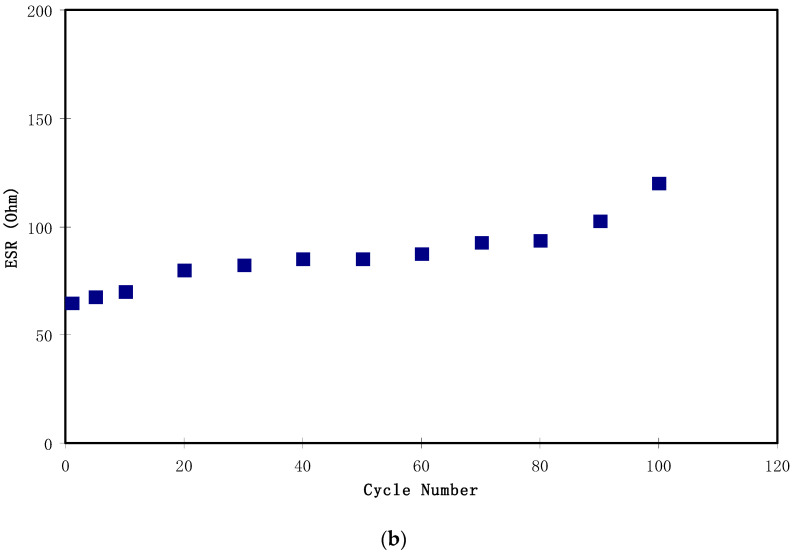
(**a**) Efficiency of the assembled EDLC for 100 cycles and (**b**) internal resistance of the assembled EDLC for 100 cycles.

Another two important parameters are efficiency (*η*) and equivalent series resistance (ESR), which were determined for the assembled EDLC in 100 cycles. The *η* value can be computed easily from the discharging (*t_dis_*) and charging (*t_cha_*) times, as shown in Figure 13a using the following equation:

(12) $$\eta =\frac{t_{dis}}{t_{cha}}\times 100$$

From Figure 13a, one can see clearly that the *η* in the 1st cycle was 43% and increased remarkably to 75% in the 5th cycle. The lower value of recorded *η* in the initial cycle might owe to the longer duration of the charging process, where the ions and electrons built the charge double layer at the surface of the carbon electrodes/electrolyte. Almost the maximum value of 92% was reached in the 30th cycle, which then became constant at an average of 92.1% up to the 80th cycle. At the steady state, charging time was almost the same as the discharging time, which is ideal for a typical capacitor. Interestingly, the value of *η* decreased to 91.5% and 90.4% in the 90th and 100th cycles, respectively. The efficiency decline of the EDLC system resulted from the development of internal resistance. It is worth mentioning that *η* was observed to be harmonized with *C_s_*, when it lowered from 65.8 F/g to 90.1 F/g in the 90th and 100th cycles, respectively. Shukur et al. [58] pointed out that a satisfactory EDLC must have 90–95% *η*. It was also claimed that a relatively high value of efficiency reflects a compatible electrolyte–electrode contact.

Figure 12a showed that the drop voltage (*V_drop_*) in the charge–discharge profile starts insignificantly prior to the discharging process starting in the assembled EDLC system. This *V_drop_* of the assembled EDLC in the present study was quantized, ranging from 0.026 to 0.048 V. This drop in potential might be related to the development of internal resistance in the bulk electrolyte, which is called equivalent series resistance (*ESR*) and can be calculated from the following relationship:

(13) $$ESR=\frac{V_{drop}}{i_{}}$$

The equivalent series resistance (*ESR*) of the EDLC for the complete 100 cycles is exhibited in Figure 13b. From the figure, the value of *ESR* in the 1st cycle was 65 Ω and increased to 75 Ω in the 10th cycle. One of the interesting observations is an almost constant value of *ESR* from the 20th to the 80th cycle, with an average value of 86.6 Ω. Furthermore, the ESR value increased to 102 Ω and 120 Ω as the cycle number was increased to 90 and 100, respectively. A harmonized trend is seen in the pattern of *C_s_* and *η*, where it is almost constant from the 20th cycle to the 80th cycle and starts to drop at the 90th cycle. Herein, three main factors are discussed that caused this increase in the internal resistance of the EDLC. Firstly, ion aggregation was formed from a rapid charge–discharge process. Secondly, the electrode–electrolyte gap resulted in an increase in the internal resistance. Thirdly, there was a technical issue caused by the fabrication of carbon electrodes on the aluminum current collector [59].

The crucial parameters in the investigation of an EDLC are energy (*E_den_*) and power (*P_den_*) density. The energy density (Wh/kg) shows how much energy can be stored by an EDLC, whereas power density (W/kg) is a measure of the energy or power that can be delivered by an EDLC [60]. Simply, both *E_den_* and *P_den_* can be obtained from the equations shown below:

(14) $$E_{den}=\frac{C_{s}V}{2}$$

(15) $$P_{des}=\frac{V^{2}}{4m\left(ESR\right)}$$

**Figure 14:**
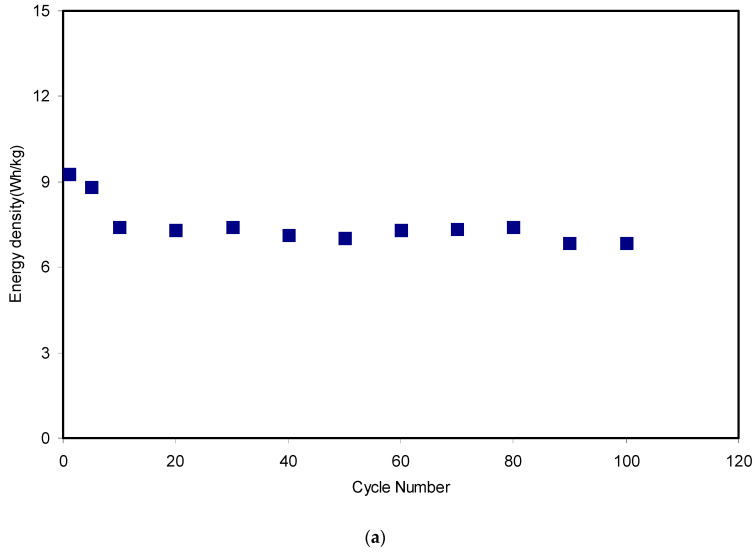

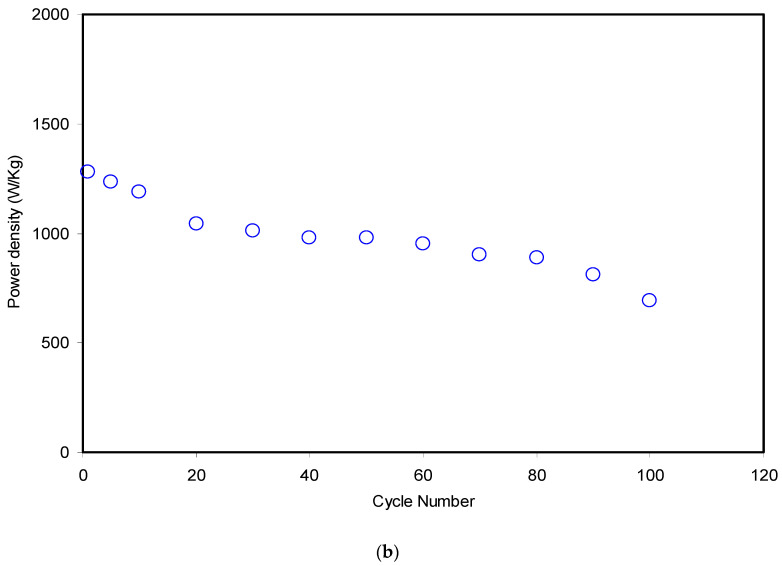
(**a**) Energy density of the assembled EDLC for 100 cycles and (**b**) power density of the assembled EDLC for 100 cycles.

Figure 14a,b presents the energy density and power density of the fabricated EDLC for 100 cycles, respectively. The applied voltage (V) on the assembled EDLC in the present work was 0.9 V and the magnitudes of both *E_den_* and *P_den_* in the 1st cycle were 9.3 Wh/kg and 1282 W/kg, respectively. These magnitudes dropped to 7.3 Wh/kg and 1041 W/kg at the 20th cycle for the energy density and power density, respectively. From the data analysis, it was recorded that the energy density magnitude was almost constant at an average of 7.3 Wh/kg and the power density magnitude remained nearly constant at 964 W/kg from the 20th cycle to the 80th cycle. The key observation is that the magnitudes of energy and power density exhibited a significant lowering in the 90th and 100th cycles.

These patterns harmonized with the patterns of *C_s_*, *η* and ESR. From an earlier study [18], the EDLC of the CS:dextran:NH_4_F electrolyte as the electrode separator reached magnitudes of 1.4 Wh/kg and 428 W/kg for *E_den_* and *P_den_*, respectively. The higher magnitudes of both the energy and power density recorded for the current work might be due to the lower extent of crystallinity of MC compared to dextran. In other words, the high ion mobility governs these crucial parameters. Furthermore, even from other studies, the degree of the crystallinity of dextran was reported to be 35.92 [61], while for MC, it was 32.89 [62]. It emphasized to a large extent that the conduction of ions is favorable in the amorphous region [63]. Ultimately, it is realized that the ion conduction in the CS:MC system is greater than in the CS:dextran host towards the electrode surface. Therefore, the more ions that diffuse and migrate towards the electrode surface, the larger the development of charge double layers, which in turn results in higher energy storage, and better performance of the EDLC. A consistent value of energy density during the ion transport towards the carbon electrodes confirms that ions in the CS:MC:NH_4_F electrolyte experience the same energy barrier [64]. Generally, the EDLC performance decline based on *C_s_*, *η*, *E_den_* and *P_den_* magnitudes is due to electrolyte depletion. It is worth mentioning that the rapid charge–discharge process causes free ions to recombine back to from aggregated ions, and consequently electrolyte depletion takes place. Eventually, the depletion of electrolyte phenomenon lowers the potential energy growth at the surface of the carbon electrodes [65,66,67,68,69,70].

## 4. Conclusions

In this work, a solid polymer blend electrolyte (SPBE), based on CS and MS incorporated with different amounts of NH_4_F, was prepared. The possibility of employing the prepared SPBE in EDLC fabrication was investigated and the performance of the device was analyzed. The FTIR spectra revealed that there was a strong interaction between CS, MS and even NH_4_F as a dopant. Both the peak shifting and intensity reduction of the XRD pattern were a good indication of the interaction between the components of the system. The structural analysis of the system confirmed the prevalence of an amorphous region. From EIS, the improvement of conductivity with increasing salt concentration was highlighted due to a rise in the number of mobile charge carriers. The charge carrying in total conduction mainly depended on ions, whereas electrons were a less common contributor, as *t_e_* and *t_i_* were determined to be 0.27 and 0.73, respectively. The rectangular shape of the CV revealed the presence of pure EDLC with capacitive behavior and recorded a high specific capacitance of 64.1 F/g. The high efficiency (above 90%) proved a good electrode–electrolyte contact. The suitability of the EDLC system was justified via energy and power density analysis. The relatively low value (86.6 Ω) of equivalent series resistance (ESR) over 80 cycles supported the good performance of the fabricated EDLC. Overall, the high conduction and electrochemical stability are behind the obtained relatively high energy and power density of 7.3 Wh/kg and 964 W/kg, respectively. Finally, it was concluded that the high conduction, electrochemical stability, low equivalent series resistance and amorphous nature played crucial roles in the good performance of the EDLC cell.

## Figures and Tables

**Table 1:** DC conductivity for pure CS:MC and blend electrolyte films at room temperature.

Sample Designation	DC Conductivity (S cm^−1^)
CMCF1	8.37 × 10^−10^
CMCF1	7.42 × 10^−7^
CMCF2	2.96 × 10^−5^
CMCF3	4.45 × 10^−4^
CMCF4	2.96 × 10^−3^

**Table 2:** The parameters of the circuit elements of the blend electrolytes.

Electrolytes	*p*_1_ (rad)	*p*_2_ (rad)	*k*_1_ (F^−1^)	*k*_2_ (F^−1^)	*C*_1_ (F)	*C*_2_ (F)
CMCF1	0.89	0.60	7.00 × 10^9^	2.90 × 10^6^	1.43 × 10^−10^	3.45 × 10^−7^
CMCF2	0.56	0.77	4.00 × 10^6^	2.55 × 10^6^	2.50 × 10^−7^	3.92 × 10^−7^
CMCF3	-	0.77	-	2.10 × 10^5^	-	4.76 × 10^−6^
CMCF4	-	0.81	-	1.13 × 10^5^	-	8.85 × 10^−6^

*k* is the inverse of *C* (*k* = 1/*C*).

**Table 3:** EDLC studies with various active materials and their specific capacitances.

System	Active Materials	*C_s_* (F/g)	Reference
CS:poly(ethylene oxide) (PEO):NH_4_SCN	Activated carbon	3.8	[55]
Poly(vinyl alcohol)(PVA):dextran:NH_4_I	Activated carbon	4.2	[56]
CS: MC:NH_4_I	Activated carbon	6.9	[8]
CS:Dextran:NH_4_I	Activated carbon	19.1	[10]
Hydroxylethyl cellulose + MgTf_2_ + EMIMT + silica nanoparticles	Activated carbon	25.1	[1]
PVA + CH_3_COONH_4_ + BmImCl	Activated carbon	31.3	[3]
MC + NH_4_NO_3_ + PEG	PEG/Activated carbon	38	[50]
PVA/polystyrene	Carbon	40	[57]
Cellulose + Na_2_SO_4_	Cellulose nanofiber + graphite	43	[21]
**CS/MC + NH_4_F**	**Activated carbon**	**64.1**	**This work**
